# A Diagnosis or a Trap: Exploring the Psychosocial and Ethical Impacts of Autosomal Dominant Polycystic Kidney Disease Diagnosis

**DOI:** 10.3390/healthcare13111316

**Published:** 2025-06-02

**Authors:** Mert Türk, Cuma Bülent Gül

**Affiliations:** 1 Department of Internal Medicine, Bursa Yuksek Ihtisas Training and Research Hospital, Bursa Uludag University, 16350 Bursa, Türkiye; mert.turk@saglik.gov.tr; 2 Department of Nephrology, Uludag University Medical School, 16059 Bursa, Türkiye

**Keywords:** autosomal dominant polycystic kidney disease, genetic screening, psychosocial impact, sociocultural factors, health literacy, patient awareness

## Abstract

**Objective**: This study aimed to explore the emotional, social, and ethical dimensions of early or presymptomatic diagnosis in individuals with Autosomal Dominant Polycystic Kidney Disease (ADPKD). **Methods**: A total of 118 participants diagnosed with ADPKD were recruited from a tertiary nephrology center in Türkiye. Data were collected via a 22-item structured and open-ended questionnaire. Chi-square and non-parametric statistical tests were used to assess associations between awareness, attitudes, and demographic variables. **Results**: Although only 10% of participants reported direct disadvantages from their diagnosis, such as difficulties in employment, insurance, or relationships, many voiced concerns about stigma and long-term uncertainties. Genetic awareness was significantly associated with increased likelihood of recommending family screening (*p* = 0.022), and higher educational attainment correlated with greater disease knowledge (*p* < 0.01). Despite emotional burden, 71.2% of participants reported adopting lifestyle modifications, and 79.6% expressed willingness to screen their children, though often with ethical hesitation. **Conclusions**: While early diagnosis of ADPKD may offer clinical benefits, it also introduces complex psychosocial and ethical dilemmas. These findings highlight the importance of integrating patient-centered counseling, clear communication strategies, and supportive policies to ensure informed decision making and mitigate potential harms.

## 1. Introduction

Autosomal Dominant Polycystic Kidney Disease (ADPKD) is one of the most common monogenic kidney diseases worldwide, affecting up to 12 million people globally. It is the fourth leading cause of kidney replacement therapy (KRT) across the world [1]. The condition is progressive, characterized by the development of kidney cysts that increase in number and size over time, eventually leading to decreased kidney function and, in many cases, end-stage kidney disease (ESKD) [2]. Diagnosis of ADPKD is usually based on family history, ultrasonography, CT, or MRI [3]. Despite advances in imaging and genetic diagnostics, there is currently no definitive cure for ADPKD [4]. Disease-modifying treatments such as tolvaptan are limited to specific subgroups of patients with rapid progression [1]. As such, the utility of early diagnosis remains ethically and psychosocially complex.

While early diagnosis may help with long-term care planning, organ donor identification, and family planning, the absence of widely effective treatment and the lifelong nature of the disease often raises the question: Does diagnosis bring empowerment or unintended harm? Individuals diagnosed presymptomatically may encounter challenges such as limited access to life insurance or employment, and often face psychological distress related to uncertainty, stigma, or future planning [2].

Genetic counseling has emerged as a cornerstone of ethical decision making in ADPKD, particularly with the growing accessibility of genetic testing. According to the updated KDIGO 2025 guidelines, genetic counseling should be provided both before and after testing, especially when diagnosis occurs in a presymptomatic phase. This process is essential for supporting informed decisions, addressing family planning concerns, and minimizing psychological distress that may arise from early knowledge of a progressive disease [1]. Furthermore, it helps patients and their families navigate the broader psychosocial implications, including insurability, employability, and stigma.

Beyond the ethical challenges, ADPKD is increasingly recognized as a condition with a substantial psychosocial burden. Recent studies have highlighted the emotional toll, social stigma, and ethical dilemmas faced by patients, including uncertainty around family planning, concerns about disclosure, and employment or insurance discrimination [5,6]. These experiences are particularly prominent in younger patients and those with a known family history. Incorporating these dimensions into research and care is essential to fully understand the impact of early diagnosis and to guide ethically sound and emotionally sensitive clinical practice.

This study aims to explore the lived experiences of individuals diagnosed with ADPKD, with a particular focus on the psychosocial and ethical consequences of early diagnosis. We sought to understand the challenges participants encountered in various aspects of life, including insurance, employment, and marriage, as well as the psychological impact of carrying a diagnosis for which no definitive treatment exists. Furthermore, we investigated whether participants would choose to undergo testing again or recommend it to their children and how perceptions of the disease and early diagnosis varied across different ages and educational levels.

## 2. Materials and Methods

### 2.1. Study Design and Participants

Participants were recruited from among patients with a confirmed diagnosis of Autosomal Dominant Polycystic Kidney Disease (ADPKD) who were either attending nephrology outpatient clinics or receiving inpatient care at the Health Sciences University Bursa Yuksek Ihtisas Training and Research Hospital. There was no prior relationship between the researchers and the patients.

Eligibility criteria included being over 18 years of age, having a definitive diagnosis of ADPKD, being a citizen of the Republic of Türkiye, and providing informed consent. Individuals were excluded if they had not yet received a confirmed diagnosis (e.g., still in the screening process), were under the age of 18, unable to read or write, or had a mental disability preventing them from participating. Non-citizens of Türkiye were also excluded.

A purposive sampling strategy was employed to capture diverse perspectives across age groups, marital statuses, and educational levels, enhancing the depth and variability of the data. This sampling approach was chosen to ensure the inclusion of individuals with potentially different life experiences, social contexts, and perceptions of early diagnosis. As this was a qualitative study, the aim was not statistical representativeness but rather to explore the range of experiences and insights relevant to the study objectives.

### 2.2. Data Collection Tool

A 22-item questionnaire (see Appendix A) was developed by the researchers based on a literature review and the specific aims of the study. The questionnaire included both multiple-choice and open-ended questions designed to assess participants’ demographic characteristics, past and current medical history, knowledge about the disease, emotional responses to diagnosis, and perspectives on family screening. Several items in the questionnaire were designed as open-ended questions to capture participants’ own words and emotional expressions. These responses provided qualitative insights into the lived experience of diagnosis and were later used as illustrative data points during analysis.

Topics covered included age, marital and parental status, educational level, time and source of diagnosis, family history of ADPKD, level of knowledge about disease progression and inheritance, as well as attitudes toward informing partners or children. Questions also explored perceived difficulties in life domains such as employment, health insurance, and marriage following diagnosis.

Prior to data collection, the draft version of the questionnaire was piloted with 10 patients to assess clarity, relevance, and flow. Feedback from these participants informed several wording adjustments, particularly for emotionally sensitive questions related to stigma and family dynamics. This pilot testing process aimed to ensure face validity and improve the user experience for the main study population.

### 2.3. Ethical Considerations

This study was conducted at the Health Sciences University Bursa Yuksek Ihtisas Training and Research Hospital, following approval from the Clinical Research Ethics Committee (Decision No: 2024-TBEK 2024/02-09, dated 21 February 2024). All participants were informed about the study’s purpose, procedures, and their rights, and both verbal and written informed consent were obtained prior to data collection. Participation was entirely voluntary, and confidentiality and anonymity were assured throughout the research process. The study adhered to the ethical principles outlined in the Declaration of Helsinki.

### 2.4. Data Analysis

Statistical analysis was performed using IBM SPSS Statistics Version 28.0. Since the variables did not meet normal distribution assumptions, non-parametric tests were applied. Distribution characteristics were evaluated using histograms, Q-Q plots, skewness/kurtosis coefficients, and normality tests. The analysis included Kruskal–Wallis tests, Cronbach’s alpha, and confirmatory factor analysis. Chi-square tests were used to examine associations between categorical variables such as demographic characteristics and attitudes toward screening and diagnosis. Statistical significance was set at *p* < 0.05.

In addition to quantitative analyses, we conducted an inductive thematic analysis of open-ended survey responses. This process followed Braun and Clarke’s six-phase framework. Two researchers independently reviewed and manually coded the qualitative data. Initial codes were generated, compared, and synthesized into themes through iterative discussion. Disagreements were resolved by consensus. Although inter-coder agreement was not formally calculated, this collaborative process ensured interpretive consistency and analytical rigor. To preserve the authenticity of participants’ perspectives and enhance thematic transparency, selected quotations from open-ended responses were presented verbatim, in accordance with qualitative research reporting standards.

## 3. Results

### 3.1. Participant Characteristics

A total of 118 individuals diagnosed with autosomal dominant polycystic kidney disease (ADPKD) participated in the study. Among them, 67 (56.7%) were female. Participants’ ages ranged from 19 to 82 years, with a mean age of 50.42 ± 14.2 years. The number of children per participant ranged from zero to six, with an average of two children. The majority of participants were married (n = 97, 82.2%), while 21 (17.8%) were single. Regarding educational background, 54 participants (45.8%) had completed primary education, 17 (14.4%) lower secondary education (middle school), 26 (22%) upper secondary education (high school), 14 (11.9%) undergraduate education (bachelor’s degree), and 4 participants (3.4%) had received postgraduate education (master’s or doctorate degree). Additionally, three individuals (2.5%) had no formal education.

### 3.2. Age at Diagnosis and Family History of ADPKD

The participants reported a wide range of ages at which they received their ADPKD diagnosis. A small proportion (13.6%) were diagnosed during childhood (0–18 years), while 17.8% received their diagnosis in early adulthood (19–29 years). The majority (50.8%) were diagnosed in middle age (30–49 years), and another 17.8% in later adulthood (50 years and above). In terms of familial history, most participants were aware of relatives affected by ADPKD, often spanning several degrees of kinship. Specifically, 15 participants had knowledge of cases in fourth-degree relatives (e.g., cousins, great-nieces/nephews), 48 in third-degree relatives (e.g., aunts, uncles, nieces/nephews), and 31 in second-degree relatives (e.g., siblings, grandparents, grandchildren). Thirteen individuals reported ADPKD only in first-degree relatives (e.g., parents, children), while just 11 participants (9.3%) stated that no one in their family had been diagnosed. Overall, 90.7% of the sample identified at least one family member with the condition, highlighting the intergenerational nature and awareness of the disease.

### 3.3. Educational Level and Disease Awareness

Participants’ awareness of the genetic and clinical nature of ADPKD varied significantly with educational level (see Table 1). When asked whether they knew that ADPKD has a 50% chance of being passed on to offspring, 87 participants (73.7%) reported awareness, while 31 (26.3%) were unaware. Similarly, 99 participants (83.9%) knew that the disease could progress to end-stage kidney disease (ESKD) requiring dialysis, whereas 19 participants (16.1%) did not.

To explore whether educational attainment was associated with disease knowledge, participants were categorized as having either below high school education (n = 74) or high school and above (n = 44). Awareness of ESKD and dialysis risk showed a statistically significant association with education: 24% of participants in the below high school group (n = 18) were unaware of this progression, compared to only 2% (n = 1) in the high school and above group (χ^2^ (1) = 9.932, *p* = 0.02). Participants were also asked whether they knew that ADPKD may affect organs beyond the kidneys, such as the liver, heart valves, or cerebral blood vessels. Only 46% (n = 54) reported awareness of these extrarenal manifestations, while 54% (n = 64) stated they had no knowledge of them. When stratified by educational level, 64% of high school and above participants were aware of extrarenal involvement, compared to just 35% of those with lower education—a statistically significant difference (χ^2^ (1) = 9.031, *p* = 0.003).

Additionally, knowledge about available medical treatments (e.g., ACE inhibitors and tolvaptan) was assessed. While 52 participants (44%) reported being familiar with such treatments, 66 (56%) had never heard of them. Again, awareness was higher among participants with high school or higher education (68%) compared to those with less formal education (36%) (χ^2^ (1) = 10.900, *p* = 0.001).

### 3.4. Age and Disease Awareness

Although one might expect disease awareness to increase with age, the findings from this study suggest a more complex relationship. Participants aged 30–49 consistently demonstrated the highest levels of awareness across various aspects of ADPKD, including its genetic inheritance, risk of progression to end-stage kidney disease (ESKD), extrarenal manifestations, and available treatment options. In contrast, the youngest age group (18–29) had notably lower levels of awareness in all domains except for ESKD progression, where their awareness was surprisingly high (100%).

Participants aged 50 and above showed a modest decline in awareness compared to the middle-aged group, particularly regarding extrarenal involvement and treatment knowledge. Despite these variations, the only statistically significant difference across age groups was observed in family screening behavior (*p* = 0.042), shown in Table 2.

### 3.5. Initiation of Family Screening: Who Makes the Recommendation?

Among the 118 participants, 70 reported that at least one relative had undergone screening for ADPKD. When asked who recommended the screening, the majority (71%) indicated that it was suggested by their physician. A smaller portion (21%) stated that they initiated screening on their own, while 8% reported that it was prompted by family members. These findings suggest that clinical guidance plays a central role in motivating patients to extend screening to family members, underscoring the importance of physician involvement in familial risk communication.

### 3.6. The Role of Genetic Awareness in Family Screening Behavior

Neither educational level nor age group was significantly associated with the likelihood of initiating family screening. However, knowledge about the genetic inheritance of Autosomal Dominant Polycystic Kidney Disease (ADPKD) emerged as a crucial factor. Among those who understood the disease’s 50% transmission risk, 66% took steps to facilitate screening for their family members, while only 42% of those without this knowledge did the same. This difference was statistically significant (χ^2^ (1) = 5.287, *p* = 0.022). In contrast, no significant association was found between awareness of disease progression to end-stage kidney disease and the likelihood of initiating family screening (χ^2^ (1) = 1.945, *p* = 0.163). These findings suggest that increasing genetic literacy among patients may positively influence the uptake of family screening, emphasizing the importance of effective patient education at the time of diagnosis.

### 3.7. Research Behavior on the Internet and Social Media

Most participants (61.9%) reported that they had not conducted any research about ADPKD through the internet or social media. As shown in Table 3, the tendency to seek information was significantly associated with both educational level and age. Among participants with an education level below high school, 72% had not searched for online resources, while only 45% of those with higher education reported the same (χ^2^ (1) = 8.008, *p* = 0.001). Additionally, older participants were less likely to conduct online searches: 73% of individuals aged 50 and above stated they had not sought information online, in contrast to only 31% of those aged 14–29 (χ^2^ (2) = 10.308, *p* = 0.006).

In addition to these quantitative findings, seven participants provided open-ended responses about their experiences seeking information online (see Table 4). These narratives revealed a wide range of emotional and cognitive responses—some participants felt more informed and reassured after learning that disease progression could be slow, while others reported increased fear or confusion. Information sources varied from academic databases such as PubMed to patient support groups on Instagram and Facebook. These insights highlight the differing ways in which individuals engage with and interpret health information in the digital space.

### 3.8. Lifestyle Changes and Educational Level

Of the 118 participants, 84 (71.2%) reported implementing at least one lifestyle change following their diagnosis of ADPKD. Reported changes included reducing salt intake, increasing water consumption, adopting a healthier diet, engaging in regular physical activity, and limiting or ceasing the use of tobacco and alcohol. In contrast, 34 participants (28.8%) indicated that they had not made any lifestyle modifications. When analyzed by educational level, no statistically significant association was found between education and the likelihood of adopting lifestyle changes (χ^2^ (1) = 2.390, *p* = 0.122).

### 3.9. Impact of ADPKD-Related Life Challenges on the Willingness to Screen First-Degree Relatives

Twelve participants (10%) reported having faced personal or professional challenges as a result of their ADPKD diagnosis. These included difficulties in continuing employment (n = 7), securing a job (n = 2), completing military service (n = 1), obtaining private health insurance (n = 1), and, in one instance, the termination of a romantic relationship. Despite these experiences, when asked whether they would still recommend family screening even in light of potential future disadvantages, 10 of the 12 individuals (83%) responded affirmatively, while 2 (17%) said they would not. Among those who had not reported such challenges (n = 106), 91 (86%) stated they would recommend screening, 7 (6.5%) would not, and 8 (7.5%) remained undecided. As shown in Table 5, the difference between the groups was not statistically significant (χ^2^ (2) = 2.321, *p* = 0.313).

Participants who answered “yes” to the question, “Would you currently choose to have your children screened for ADPKD?” were asked to explain their reasoning in an open-ended format. Responses were collected verbatim and are presented in Table 4. These reflect a wide range of motivations and concerns, illustrating the diversity of attitudes toward early diagnosis in children.

### 3.10. Pre-Marital Awareness and Disclosure Behavior in ADPKD Patients

Among the 101 participants who were currently or previously married, 80 (80%) reported being unaware of their ADPKD diagnosis prior to marriage. Of the 21 participants who knew about their diagnosis before marriage, 12 disclosed this information to their partner, while 6 chose not to, and 3 did not provide a response. Among the 21 unmarried participants, 15 (71%) indicated that they would inform a future partner about their diagnosis. One unmarried participant stated that they would not disclose their diagnosis, another was undecided, and four reported no plans to marry and did not respond to this question. Additionally, there was no significant association found between knowledge of the genetic inheritance of the disease and the willingness to disclose the diagnosis to a partner (χ^2^ (2) = 0.646, *p* = 0.724).

### 3.11. Perceptions of Future Risks for Diagnosed Children

Participants were asked what types of challenges they believed their children might face if diagnosed with ADPKD. Their open-ended responses are presented verbatim in Table 4. Concerns ranged from social stigma and relationship difficulties to emotional strain, employment concerns, and access to health services.

## 4. Discussion

This study examined the psychosocial and ethical dimensions of being diagnosed with Autosomal Dominant Polycystic Kidney Disease (ADPKD), particularly in the context of presymptomatic testing. While early diagnosis can provide some clinical benefits, such as enabling blood pressure control, promoting lifestyle changes, or initiating disease-modifying treatments like tolvaptan, many patients questioned its overall value. They emphasized the unpredictable nature of the disease and expressed skepticism regarding the extent to which early interventions can alter long-term outcomes. Our findings indicate that although early diagnosis allows for closer monitoring and future planning, it can also result in emotional distress, social disadvantages, and practical challenges for both individuals and their families. To improve conceptual clarity, we distinguish between psychosocial consequences, such as emotional distress and stigma, and structural barriers like employment or insurance discrimination, which affect patients in different ways.

Participants frequently expressed concerns not only about the medical implications of early diagnosis but also about its broader psychosocial consequences. Many emphasized fears related to social stigma, particularly in employment, health insurance, and marital relationships. Although only 10% had experienced direct disadvantages, a significantly larger portion anticipated such outcomes. These concerns are consistent with the literature on presymptomatic testing for hereditary diseases, where reduced autonomy, increased anxiety, and fear of discrimination are commonly reported [7,8]. Clarke et al. similarly observed that many individuals avoid or postpone genetic testing, not because of clinical concerns, but due to the potential social disruption such a diagnosis may cause [9]

The desire to screen children for ADPKD was notably high in our sample, with 79.6% of participants expressing strong interest. Most cited a sense of responsibility to guide medical care early. However, open-ended responses revealed emotional ambivalence; while many believed early diagnosis would benefit their children, some raised concerns about labeling them with a lifelong, incurable disease. One participant stated, “They might have a childhood as unhappy as mine... Taking medication all the time is hard... Everyone will always ask, ‘What’s wrong?’” (P.14, female, 22). This dilemma—whether knowledge empowers or harms—is well-documented in the literature [10]. Domínguez et al. and Clarke et al. similarly noted that patients often defer testing or disclosure to preserve their children’s psychological well-being and autonomy [8,9].

Marital and relational challenges were also prominent in our findings. Among participants diagnosed before marriage, some chose not to disclose their condition to their partners due to concerns about rejection, perceived stigma, or jeopardizing the relationship. While 71% of unmarried participants stated they would share such information with a future spouse, several expressed hesitation and emotional uncertainty. These dilemmas align with previous research suggesting that individuals carrying genetic risk may fear being perceived as a “risky partner”, potentially facing moral judgment or relationship instability [9,11,12,13]. Disclosure is often delayed or softened to protect loved ones, maintain relational harmony, or avoid being labeled as genetically flawed.

Our data also highlights the influence of education on disease awareness and digital health-seeking behavior. Participants with higher education levels were significantly more likely to access health information online. While some found reassurance through academic resources and social media groups, others experienced heightened anxiety, especially when confronted with unfiltered stories of dialysis or transplantation. Domínguez et al. and Ebrahimi et al. emphasized that the unpredictable nature of disease progression and the absence of curative treatment often result in psychological distress, particularly in the early period following diagnosis [8,14].

Awareness of disease progression, heritability, and treatment options was higher among younger and more educated individuals. Participants with at least a high school education were significantly more likely to be aware of the disease’s potential to progress to end-stage kidney disease, the 50% heritability risk, and the availability of ACE inhibitors or tolvaptan. These findings are consistent with Dogan et al., who found lower awareness scores in older and asymptomatic patients [15]. Contributing factors may include reduced health literacy, fewer healthcare interactions, and lower motivation for disease education.

Despite the psychological burden of an ADPKD diagnosis, most participants reported making positive lifestyle changes, such as increasing water intake, reducing salt, improving dietary habits, and limiting alcohol or tobacco. Interestingly, no significant association was found between education level and these changes. This contrasts with health behavior models that typically predict better adherence among more educated individuals, suggesting that in ADPKD, intrinsic motivation and lived experiences may be stronger drivers of behavior change [16]. Family members already affected by the disease may also influence health behaviors in newly diagnosed individuals. These findings highlight the need to integrate psychosocial support and genetic counseling into routine ADPKD care, particularly in healthcare systems where structural protections may be limited.

### Limitations

This study has several limitations. First, it was conducted at a single tertiary care center in Türkiye, which may limit the generalizability of the findings. Second, although the inclusion of open-ended questions provided valuable qualitative insights, the clarity and depth of responses varied across participants. Third, some items (e.g., stigma, reproductive decisions, and disclosure) were emotionally sensitive, which may have introduced recall or response bias due to social desirability. Nevertheless, combining quantitative analysis with verbatim narratives strengthened the contextual interpretation of the findings. Fourth, the study did not include a comparison group, such as late-diagnosed individuals or patients with other hereditary conditions, which could have further contextualized the psychosocial findings. Finally, although the questionnaire underwent pilot testing and expert review for content validity, no formal validation procedures (e.g., construct validity or reliability testing) were conducted, consistent with the exploratory nature of the study.

## 5. Conclusions

The findings of this study demonstrate that a diagnosis of ADPKD carries significant psychosocial, relational, and ethical implications that extend well beyond clinical management. While early detection may offer opportunities for medical intervention, it can also introduce emotional distress, social stigma, and uncertainty, particularly in the absence of a definitive cure. These findings may inform clinical practices related to genetic counseling, patient education, and shared decision-making, particularly in younger individuals and those undergoing presymptomatic testing. Therefore, presymptomatic genetic testing should be accompanied by comprehensive genetic counseling, psychosocial support, and safeguards against discrimination. In answering the central question posed by this study— “Is it a diagnosis or a trap?”—our findings suggest it may be both: a gateway to early awareness and preparedness, but also a source of vulnerability if not ethically and contextually managed.

## Figures and Tables

**Table 1:** Disease awareness by educational level.

Knowledge/Behavior	Below High School (n = 74)	High School and Above (n = 44)	χ^2^ (df)	*p*-Value
Aware of genetic transmission	66% (n = 49)	86% (n = 38)	5.89 (1)	0.015
Aware of progression to ESKD	76% (n = 56)	98% (n = 43)	9.93 (1)	0.020
Aware of extrarenal involvement	35% (n = 26)	64% (n = 28)	9.03 (1)	0.003
Aware of treatment options	36% (n = 27)	68% (n = 30)	10.90 (1)	0.001
Family members screened	67% (n = 47)	52% (n = 23)	1.44 (1)	0.229

ESKD = end-stage kidney disease. χ^2^ = chi-square test statistic; df = degrees of freedom.

**Table 2:** Disease awareness by age group.

Knowledge/Behavior	18–29 (n = 21)	30–49 (n = 60)	50+ (n = 37)	*p*-Value
Aware of genetic transmission	48% (n = 10)	80% (n = 48)	78% (n = 29)	0.330
Aware of progression to ESKD	62% (n = 13)	93% (n = 56)	81% (n = 30)	0.246
Aware of extrarenal involvement	29% (n = 6)	57% (n = 34)	38% (n = 14)	0.817
Aware of treatment options	19% (n = 4)	57% (n = 34)	39% (n = 14)	0.407
Family members screened	33% (n = 7)	60% (n = 36)	62% (n = 23)	0.042 *

ESKD = end-stage kidney disease. Analysis was performed using the chi-square (χ^2^) test; * for “Family members screened”: χ^2^ (2) = 6.31, *p* = 0.042.

**Table 3:** Internet and social media research by education and age group.

Variable	Below High School	High School and Above	Age 14–29	Age 30–49	Age 50+
Searched Internet/social media	21 (28%)	24 (55%)	9 (69%)	18 (48%)	18 (27%)
Did Not Search	53 (72%)	20 (45%)	4 (31%)	20 (52%)	49 (73%)

Note: Chi-square tests indicated significant associations between education level and information-seeking behavior (χ^2^ (1) = 8.01, *p* = 0.001), and between age group and information-seeking behavior (χ^2^ (2) = 10.31, *p* = 0.006).

**Table 4:** Selected patient quotations from open-ended survey items.

Participant (Initials and Age)	Response
Question: *After diagnosis, did you search for information on the Internet or social media? If so, how did it affect your perception of the disease?*	
K.Ç. (46)	I found out there is no cure, realized how serious the disease is, looked up all the professors who deal with it, and learned that there’s no surgery option.
A.M. (22)	Of course I searched online. I focused more on the psychology of being a patient. I looked up medical articles. I used PubMed rather than social media. The psychological aspect was just as important to me as the medical one.
G.H. (71)	I searched online and found out it wasn’t as scary as I thought. It turns out I don’t have to go on dialysis immediately. I realized I need to go to my check-ups regularly.
İ.U. (34)	I did research online. It really changed my perspective. I found out kidney failure might appear at a much later age, and that comforted me. I also learned it’s a common disease but not well-known.
E.Y. (46)	I searched on social media. There are groups for polycystic kidney disease patients on Instagram and Facebook. I joined those. Some are on dialysis, some do peritoneal dialysis, others have had transplants. I read their life stories.
N.Ç. (44)	What I found online scared me even more.
H.Ş. (24)	I did research. I didn’t go on social media. I used medical journals. I even had English articles translated into Turkish. It was satisfying. I didn’t find anything different from what my doctors had told me.
Question: *Regardless of previous screening, would you currently have your children screened? If yes, why?*	
F.Ç. (62)	My children tested negative, but I will have my grandchildren tested. I’m on peritoneal dialysis nine hours a day. I only live to dialyze. Yes, I may be eligible for a transplant, but I can’t risk my loved ones. My children and grandchildren constantly see me hooked up at home. If they also have the disease in the future, they will constantly have to go to the hospital. Besides all that, knowing you will eventually end up on dialysis is devastating.
A.M. (22)	This is where ethics comes in. Normally, I’d want to ask my child’s opinion, but I think I would ignore that and have the screening done anyway.
H.A. (46)	Absolutely. Screening must be done. I started dialysis at an early age. I’d have them screened so they’d be careful. I’ll even have my grandchildren screened.
M.U. (61)	I had a kidney transplant. Many in my family are on dialysis or waiting for a transplant. My children will take precautions if they know early. There won’t be a lingering “what if” in their minds. I even had my 8-year-old grandchild tested—it was negative. But I’ll do it again when they’re older. You can recover from the shock of an early diagnosis, but you can’t recover from dialysis. Whatever obstacles they might face, I still went through with the screening.
Question:* If your children are diagnosed with the disease, what kinds of problems do you think they might face in the future?*	
N.M. (19)	They won’t be able to fully experience anything in life. They may face problems getting married, struggle to find a partner. Society might stigmatize them. People might say, “They can’t have children,” or “They’ll pass on the disease”.
D.E. (22)	They’ll get sick often and face social exclusion. In gym class, they’ll be treated differently. Everyone will always ask, “What’s wrong?” There might be constant risk of infection. Taking medication all the time is hard. Feeding a child with this condition can be challenging. They might have a childhood as unhappy as mine.
R.İ. (49)	I don’t think they’ll have any problems. So far, they haven’t even known they had it. Nothing bad has happened. Knowing won’t change anything.
E.Y. (46)	Fear of dialysis might lead to low self-esteem. I was scared as a child when I saw my father on peritoneal dialysis. Other than that, I don’t think they’ll face major problems with insurance or employment in this country.
S.B. (52)	My son has only one kidney. If the other one fails, his life could be terrible. The idea of dialysis will always be on his mind. He might hesitate to have kids, or not have any at all. If I had known, I wouldn’t have had children.

Note: All participant quotations are presented verbatim to preserve authenticity and accurately reflect the original voices of respondents, in accordance with qualitative research reporting standards.

**Table 5:** Comparison of family screening preferences based on perceived life disadvantages due to ADPKD.

Experienced Disadvantage Due to ADPKD	Would Recommend Screening (n, %)	Would Not Recommend (n, %)	Undecided (n, %)	Total
Yes (n = 12)	10 (83%)	2 (17%)	0 (0%)	12
No (n = 106)	91 (86%)	7 (6.5%)	8 (7.5%)	106

Although a high proportion of participants who had experienced disadvantages expressed willingness to screen their relatives, this difference was not statistically significant when compared to those without such experiences (χ^2^ (2) = 2.321, *p* = 0.313).

## Data Availability

The original contributions presented in this study are included in the article. Further inquiries can be directed to the corresponding author.

## Appendix A. Survey Questions

**Section A**: Demographic Information

1. What is your age?
2. What is your marital status?
3. Do you have any children? If yes, how many?
4. What is your highest level of education?

**Section B**: Diagnosis Experience

5. When did you learn about your ADPKD diagnosis?
6. How and from whom did you learn about your diagnosis?
7. Do any of your family members have ADPKD? If yes, who?

**Section C**: Knowledge and Awareness

8. How much do you know about ADPKD?
9. Did you know that ADPKD may lead to end-stage kidney disease and require dialysis in the future?
10. Did you know that ADPKD is a genetic disease with a 50% chance of being passed on to your children?
11. Are you aware that there is currently no cure for ADPKD, and that treatment mainly aims to slow disease progression and prevent cardiovascular complications?
12. Did you know that ADPKD can affect other organs beyond the kidneys and cause related symptoms?

**Section D**: Screening and Family Involvement

13. Have you had your family members screened for ADPKD?
14. If yes, who recommended the screening?

**Section E**: Personal Impact and Lifestyle

15. How has your lifestyle changed since receiving the diagnosis?
16. How did you feel emotionally after your diagnosis?
17. Did you search for information about the disease on the internet or social media after your diagnosis? If yes, did it satisfy your need for information and influence your perception of the disease?

**Section F**: Social and Ethical Impacts

18. Has your diagnosis caused any difficulties in obtaining health or life insurance, finding a job, or getting married? If yes, in which areas?
19. If you had known that your diagnosis might cause such challenges, would you still have undergone screening?

**Section G**: Reproductive Decisions and Future Planning

20. If you knew about your diagnosis before marriage, did you talk to your partner about it?
21. If you are not married, would you consider informing a potential partner about the hereditary nature of the disease?
22. If you have children who have not been screened, would you consider having them tested? Why or why not? What kind of challenges do you think your children might face if they receive a diagnosis?

Note:

The following questionnaire was developed specifically for this study, based on a literature review and the research aims. It includes both closed-ended and open-ended questions designed to explore participants’ demographic background, diagnostic experience, level of disease awareness, emotional responses, social challenges, and attitudes toward family screening and future planning.
